# Passing Events and Topics

**Published:** 1921-03-26

**Authors:** 


					Passing Events and Topics.
Wellcome Historical Medical Museum.
The Wellcome Historical Medical Museum will be closed
for redecoration and cleaning from April 1 to April 30
inclusive.
York County Hospital.
The York Workpeople's Hospital Fund have arranged to !
make the annual liouse-to-house collection for the County
Hospital during the week ending April 50.
London Temperance Hospital.
A special appeal for ?6,COO is being made by the London
Temperance Hospital in order to secure the ?6,000 promised,
by King Edward's Hospital Fund for a scheme of
reconstruction.
Specialists for the Poor.
The Brentford Guardians have postponed consideration
of a scheme, estimated to cost ?1,000 a year, to secure the
regular visits of specialists to consult with the medical
superintendent of the infirmary.
New Maternity Home at Swansea.
Through tho generosity of the American Red Cross
Society, which made an establishment grant of ?2,000,
Craig House, Gore Terrace, Swansea, has been transformed j
into a maternity home. Though the institution is a com- (
paratively small one (ten beds) it is thoroughly up to date j
in its equipment, ami application is being made for its
recognition as a training-school for midwives. Tho new
home was formally opened on March 11 by the Mayoress
of Swansea, Mrs. Percy Molyncux.
Information for Nurses.
The Society for the Prevention of Venereal Disease will
-supply any nurse, who sends to tho Hon. Secretary of tho
Society at 143 Harley Street, W. 1, a stamped addressed '
" Wit}1 ?TCS its " Directions for Men" ??;
anv furt1ir>li> ?f oim>u" Pamphlets, and will give thc"
may ' t n ?nnatl0n roSiarili"g" the Society thai the?
as l8SOn J' fl P,aymcnt of 2?- 6d. nurses can be enr60*
? be entitled to ft*
Maternity and Child Welfare Centres and Day
t\ !,? . Nurseries.
ijr ;7"""'-v"??W> |.? .-I rcviU iw> ?S
and W I n"d ChiM ?"''lfere Centres in K'f1""
.ml : ST,""' ,Kli"s' '?r ,l"> "me, a ? d'S
73c s" 0 llst contains tin* names and addresses
social V"' 140 <lu-v "u?"<* conducted b, volant.'?
ducted k ?/ I'183 &"ltra ""<1 ? -lay nurseries eo?
lln'e?1 ? ?, . authorities. It i., obtainable from ?,
2s net ' Stationery Office, or throuBli any booksoll".
Nurseries Needed? _..v.
where" ''iY" d,"y lmrserics aro not a long-felt want d ?
of tho St i,US *le iIilte?iity and Child Welfare Coinn\illCe
it r S.t0.ke ,Newi"gton Borough Council states that *
at thn1 thre? m?nths "P? *?>a11 flttT ?0-
metit an!'/ "!n'S0l7 Jt l>ns renewed its efforts, by oVvn
throuo'i, ' i?i ierw,se> (o make the provision bettor v0lJld
lead T ? ? l)orou^1 i? the hope that these efforts tj,e
daily 0flnCTSCd lltte,,t|ances. But, notwithstanding ^
stances' tho V'om? "ot ,mPrt,veil, and. in, ^the tJ1Vf
has arrival S "!A oxPrcssc? the opinion that _ ju
baling too t?1 I? discontinuance of the "ursCufs bcc"
small nnT'- ? attend?nce at thc Day Nursery? haj d t0
flose 'thn t"! cons?quonco the Committee has <fec? ,tf flt
the end s'x months, and only to re ffere^
for ro ' Vf thaA tlfr if at least twenty children arc <*
'ifcuinr attendance.

				

